# Marketing of Freshwater and Marine Fish Species in Alexandria City, Egypt: Human Health Risk of Specific Metals

**DOI:** 10.1007/s12011-025-04596-z

**Published:** 2025-04-17

**Authors:** Khaled A. Osman, Hala H. Elsayed Mohamed, Maher S. Salama

**Affiliations:** 1 Pesticide Chemistry and Technology Department, Faculty of Agriculture, EL-Shatby, Alexandria University, Aflaton St., EL-Shatby, 21545 Alexandria, Egypt; 2 The Faculty of Organic Agriculture, Heliopolis University, P.O. Box 2834, Cairo, Egypt

**Keywords:** Fish, Alexandria, Metals, Seasonal variations, Metal Pollution Index, Health risk

## Abstract

Fish intake may constitute a significant route by which humans are exposed to metals, especially people who depend on fish as a source of protein as Alexandrians. Therefore, this study aimed to investigate the metal contents in muscles in eight commonly consumed freshwater (Tilapia, Catfish, and Common Carp) and marine fish species (Emperors, Groupers, Mackerels, Silver Pomfret, and Roving Groupers) collected from the local markets located in Alexandria City for a 1-year calendar year, 2022. Seasonal variations in the levels of the tested metals in the fish species, with significant differences between the species, were recorded. Also, the levels of Cu, Zn, Fe, Co, and Cd in all the tested fish species collected during the experiment did not exceed the guideline limits, while Ni, Cr, and Pb in fish collected during October–May, June–September, and February–May 2022, respectively, exceeded the permissible limits settled by FAO and WHO. Additionally, Mackerels and Roving Groupers had high-level contents of Mn that exceeded the permissible limits settled by European Commission. The accumulation of these metals in muscles of different fish species had relative variation in the accumulation, and Cu was the most predominant element in Tilapia, Zn in Catfish, Roving Groupers, and Mackerels, Fe in Common Carp, Groupers, Emperors, and Silver Pomfret, and Co in Tilapia. Consumption of fish with a high Metal Pollution Index (MPI) value may pose a potential public health risk. On the meantime, the calculated values of estimated daily intakes (EDI), hazard index (HI), and target health quotient (THQ) indicated no potential health risk for Alexandrians via the consumption of these fish species because they did not exceed the World Health Organization’s acceptable daily intake.

## Introduction

Although the progress of industrialization enhances the human quality of life, it is crucial to recognize that these advancements may affect the quality of the environment [1]. Environmental pollution caused by heavy metals has aroused widespread concern around the world in the food we eat, water we drink, and environment we live in, either naturally or due to human activities such as industrial emissions, agricultural practices, or contamination during manufacturing. Anthropogenic, various industrial sources, combustion by-products, and traffic are the main sources for these metals [2–4]. Either as dissolved components in water or as an essential component of suspended sediments, metals move through rivers and streams; the latter have the highest potential for the most harmful consequences [5]. The degree of contamination will depend on how close the well is to the mining site. They may then be stored in riverbed sediments or seep into the subsurface water, contaminating water from underground sources, especially wells. As a result, it has been claimed that heavy metal concentrations in wells close to mining operations surpass drinking water standards.

Food probably represents the largest source of exposure to metals for humans, where the actual concentration of metals in food varies widely depending upon the food product, the growing conditions, and the type of processing [6]. Unfortunately, metals are persistent, nonbiodegradable, and accumulate in the aquatic environment [7, 8]. The metal effects on human health are of great interest today, especially for aquatic food products when ingested over a long time [9, 10]. The high levels of these metals can range in determinate effects, such as gastric irritation, diarrhea, kidney and liver dysfunction, cardiovascular system disorders, damage to the central and peripheral nervous systems, and affect organ systems [11]. The targets for toxicity are enzymes and/or membranes of cells and organelles and compete with respect to their absorptive sites [12–14]. Although fish is a food with health-promoting effect and valuable source for protein for human health due to their content of low saturated fat, omega-3 fatty acid, minerals, vitamins, easily digestible protein, essential amino acids, and mainly unsaturated fatty acids, fish is a food with health-promoting effect [15]; there is a risk that environmental contaminants may enter seafood products either through water and sediments or via feed and feed additives or may be occurred during fish processing and storage [16] and then enter the human body with fish consumption causing a potential hazard to human health, especially for people who consume large amounts of it [9]. The pollution caused by heavy metals, even at minimal concentrations, poses a substantial threat to seafood’s consumers [17].

Many studies were conducted in fish to monitor the food supply for toxic and nutritionally important metals in fish samples harvested from the Black and Aegean Seas, Turkey [18], Egypt [16, 19], the Oman’s Gulf [20], the Persian Gulf of Iran [21], the Portuguese coast [22], Bangladesh [8], Saudi Arabia [23], and India [24]. Human activities, natural processes, water, and sediments are responsible for the buildup of heavy metals in fish [25, 26]. These metals are divided into non-essential elements like Cd, Hg, and Pb, which have no physiological significance roles for organisms, and essential elements like Co, Cu, Fe, Mn, Mo, and Zn, which are essential for a variety of physiological and biochemical processes [27]. When their concentrations remain within the accepted limits, most of these metals are essential for fish metabolic processes; when they surpass these limits, they become very harmful to both humans and fish [28, 29].

Due to declining fish populations and rising overfishing, aquaculture projects must be developed [30, 31]. Nowadays, aquaculture projects supply over 20% of the average daily animal protein consumption for about 3.2 billion people globally and nearly 50% of all fish meant for direct human consumption [32]. Compared to other agricultural sectors that produce food derived from animals, the aquaculture sector is growing at a substantially quicker rate [33]. Significant amounts of contaminants are produced in the water as a result of the intensification of fish aquaculture [34]. To reduce the dangers to consumer health, water management must be monitored and optimized.

With a population of 102 million, Egypt’s total fish production was 2.2 million metric tons in 2021; the country has a promising strategy to increase fish production to 3 million metric tons by 2025 [35]. Fish intake per capita increased from 16.67 kg/year in 2012 to 21.60 kg/year in 2021, which is higher than the worldwide (15.3 kg) and African (10.4 kg) averages and nearly equal to the average consumption in Europe (22 kg) [36]. Unfortunately, negligible data are available on the contents of the potentially toxic and non-toxic metals in fish sold in the local market of Alexandria Governorate, Egypt. Therefore, the present study was designed to provide seasonal background information on the levels of some metals in freshwater and marine fish species collected from different local markets during January–April, May–August, and September–December 2022. Also, the present investigation was carried out to determine the possible health hazard for populations consuming fish muscles contaminated with these metals by determining the estimated dietary intake.

## Materials and Methods

### Chemicals

All chemicals used in the present study are analytical-reagent grade. Standards solutions of metals were prepared by adequate dilution of element standard (1000 mg/L) obtained from J. B. Baker Inc. (Phillipsburg, NJ, USA) in deionized water (pH 7.0) of 15 MΩ cm resistivity obtained from a water purification system (PURELAB Option-R, ELGA, UK). Nitric acid (65%) was purchased from BDH Chemical Poole, England, while H_2_O_2_ (30%) was obtained from Merck. All solutions and dilutions were prepared. Glassware was washed with detergent, rinsed with deionized water, oven dried at 130 °C, and then washed with nitric acid.

### Fish Sampling and Preparation

Freshwater fish wild-caught including, Tilapia (*Oreochromis niloticus*, Family: Cichlidae), catfish (*Clarias* spp., Family: Clariidae), Common Carp (*Cyprinus carpio*, Family: Cyprinidae), and marine fish wild-caught including, Emperors (*Lethrinus* spp., Family: Lethrinidae), Groupers (*Epinephelus* spp., Family: Serranidae), Mackerels (*Scomberomorus commerson*, Family: Scombridae), Silver Pomfret (*Pampus argenteus*, Family: Stromateidae), and Roving Groupers (*Plectropomus pessuliferus*, Family: Serranidae) were collected during January–April, May–August, and September–December 2022 (15 individuals of each species representing the size range commercially available to custom were collected every month) from the same three markets located in Alexandria City, Egypt. The sources of freshwater and marine fishes were the Nile River and the Red Sea, respectively. All fish collected were healthy and did not have any lesions on their bodies. After being cleaned with distilled water and placed in a sterile, labeled plastic bag, the samples were promptly transported to the lab in an ice box (at 4 °C). Dorsal muscles (anterior to dorsal fin) of each sample were removed, composited to obtain representative samples, packed separately, and then stored in a deep freezer at − 20 °C until analysis.

All the glassware’s were soaked in concentrated nitric acid then rinsed with ultrapure water and dried in an oven for use. The frozen fish samples were thawed at room temperature and dissected using stainless steel surgical blades. Muscle tissue was isolated from each fish sample, dried in an oven at 105 °C for 24 h (Carbolite, Thermal Engineering Services, Vietnam), grounded into powder using a grinder, and then 0.5 g of fish powder was transferred into the crucible containing a few drops of concentrated nitric acid and H_2_O_2_, covered with a lid, and then placed in a muffle furnace (Heraeus, Germany). The temperature was increased stepwise up to 550 °C and then left to ash at this temperature for 24 h [37]. Ash was left to cool at room temperature, rinsed with 1 M nitric acid, filtered into a 25 ml volumetric flask using Whatman filter paper No. 42, and then completed to the mark with 1 M nitric acid. The same method was used for digestion of blank solutions (without sample) to ensure the accuracy of the heavy metal analysis; if the blank solutions showed any residual content of heavy metals, then they must be excluded from the result of the atomic absorption spectrophotometer. A stock solution of the analyzed element with a concentration of 1000 mg/L was diluted by using ultrapure acidic water (5%, V/V HNO_3_) to make calibration curves.

### Heavy Metals Analysis

Metals concentrations were measured by using an atomic absorption spectrometer (AAS, Shimadzu Model AA-6200, Kyoto, Japan), equipped with a hollow cathode lamp of the tested metals and a 10-cm-long slot-burner head in which air and acetylene as oxidizer and fuel were used, respectively. Following the manufacturer’s regular operating procedures, the spectrometer’s working settings were adjusted. The analytical conditions for the measurement of the heavy metals in the sample using AAS are presented in Table 1. Blank solutions were prepared under identical conditions, and the average signal was subtracted from the analytical signals of fish samples. Five replicates of each sample were used to determine the mean metal value. Metals in the blank were measured and subtracted from each analyzed fish sample.

**Table 1:** Flame atomic absorption spectrophotometer operating conditions and linear working ranges for the tested metals

Element	Gas type	Flow rate (L/min)	Wavelength (nm)	Slit width (nm)	Current (mA)	Linear working range (mg/L)
Cu	Air/acetylene	1.80	324.70	0.70	6	0–2
Zn	Air/acetylene	2.00	213..90	0.70	8	0–2
Fe	Air/acetylene	2.20	248.33	0.20	12	0–10
Co	Air/acetylene	2.20	240.7	0.20	12	0–50
Ni	Air/acetylene	2.20	232.50	0.20	12	0–2
Mn	Air/acetylene	2.00	279.50	0.20	10	0–2
Cr	Air/acetylene	2.80	357.9	0.70	10	0–10
Cd	Air/acetylene	1.80	228.80	0.70	8	0–2
Pb	Air/acetylene	2.00	217.00	0.70	12	0–2

### Validation Method

The metal contents, Cu, Zn, Fe, Co, Ni, Mn, Cr, Cd, and Pb in fish samples were determined using atomic absorption spectrometry (Shimadzu Model AA-6200, Kyoto, Japan) according to the standard guidelines of the manufacturer.

For construction of calibration curves, the prepared standard solutions at concentrations of 0.0–2.0 in all the tested elements except for Fe and ranged from 0.0 to 10.0 mg/l were prepared from the stock standard solution (1000 mg/l), and the results were expressed as mg/kg of fresh fish (Table 2). The method of validation for measuring heavy metals was determined by measuring the limits of quantification (LOQ) and detection (LOD), relative standard deviation (RSD), and the recovery percentages. For validation of all analytical techniques used, dogfish liver DOLT-4 purchased from Canada was used as a certified reference material. The recovery of CRM is shown in Table 2. The LOD and LOQ limits are defined as the concentration (signal) corresponding to three and ten times the standard deviation of 10 measurements of a reagent blank (noise), respectively. Samples with metal content below detection limits were assigned a value of zero. The LOD values of elements (as mg/kg) were 0.06, 0.12, 0.17, 0.40, 0.20, 0.34, 0.10, 0.13, and 0.08, while LOQ values were 0.22, 0.40, 0.56, 1.23, 0.67, 1.02, 0.32, 0.36, and 0.30 mg/kg for Cu, Zn, Fe, Co, Ni, Mn, Cr, Cd, and Pb, respectively, with response (*R*^2^) > 0.96. The percentages of recovery were determined at levels of 0, 0.25, and 0.5 mg/g. The recoveries were 93, 105, 96, 90, 104, 100, 90, 104 and 97% for Cu, Zn, Fe, Co, Ni, Mn, Cr, Cd, and Pb, respectively, with RSD ranged from 6 to 9% (Table 2).

**Table 2:** Standard curves, limits of detection (LOD) and quantification (LOQ), % Recovery, and precision for the tested metals

Metals	Response (R^2^)	Correlation coefficient (R)	LOD (mg/kg)	LOQ (mg/kg)	% Recovery	Precision
Cu	0.996	0.998	0.06	0.22	93±6.5	6.99
Zn	0.989	0.995	0.12	0.40	105±8.0	7.62
Fe	0.996	0.998	0.17	0.56	96±9.0	9.38
Co	0.998	0.995	0.40	1.23	90±5.5	6.11
Ni	0.992	0.996	0.20	0.67	104±6.0	5.77
Mn	0.996	0.998	0.34	1.02	100±8.0	8.00
Cr	0.998	0.999	0.10	0.32	90±6.0	6.67
Cd	0.927	0.963	0.13	0.36	104±7.0	6.73
Pb	0.999	0.999	0.08	0.30	97±4.0	4.12

### Estimated Daily Intake (EDI)

The EDI values were calculated according to Eq. 1 [38]:

1 $$\text{EDI= }\frac{\text{CxIR}}{{\text{BW}}}$$

where C (μg/g) is the concentration of the element in the muscle sample, IR is the daily consumption of fish for adults, taking into account 21.60 kg/year in 2021 (59.19 g/day) [35], which is above the global (15.3 kg) and African (10.4 kg) averages [36, 39] and close to the average European consumption (22 kg). BW stands for body mass of fish consumer in Egypt aged 16–70 years [40]. The HI was calculated by comparing the EDI of each element with the acceptable daily intake (ADI) established [41, 42] as shown in Eq. 2:

2 $$HI=\frac{EDI}{ADI}\times100$$

### Target Hazard Quotient (THQ)

The non-carcinogenic health risk assessment of the multiple tested metals in fish was assessed by calculating the target hazard quotient (THQ) as in Eq. 3:

3 $$THQ=\frac{EDI}{Rfd}\times10^{-3}$$

where RfD is the corresponding reference dosage and refers to the maximum acceptable dose that can be consumed orally without likely causing any risks [43].

### Metal Pollution Index (MPI)

Metal pollution index (MPI) was assessed using the following equation (Eq. 4) [44]:

4 $$MPI=\left(M_1\times M_1\times M_1\times\dots\dots\dots\times M_1\right)^{1/n}$$

where M_1_, M_2_, M_3_ …, and M_n_ stand for the concentrations (mg/kg) of the first, second, third, and the *n*th total number of studied metals under consideration in the tissue of fish species, respectively.

### Statistical Analysis

The statistical package of the Costat Program was used for all chemometric calculations [45]. All the data were calculated as mean ± standard deviation (SD) and analyzed using a two-way analysis of variance (ANOVA) and least significant difference (LSD) multiple comparison tests were used to compare between treatments at a level of 0.05 (*p* < 0.05). The Tukey posthoc test was used to ascertain significant differences. The Pearson correlation coefficient was calculated to determine the correlations between the evaluation criteria included (across the different metals).

## Results and Discussion

### Metals Content of Different Freshwater and Marine Fish

Metals (Zn, Fe, Cu, Co, Ni, Mn, Cr, Pb, and Cd) were chosen as representative trace metal indices of environmental pollution. The results indicated varying concentrations of metals among different fish species (Tables 3, 4, 5). Three mineral groups can be differentiated: elements that are very abundant (Fe, Zn, Co, and Mn), elements in medium concentrations (Cu, Ni, Cr, Pb), and non-detected elements (Cd).

**Table 3:** Species and seasonal variations in levels (mg/kg fresh weight) of Cu, Zn, and Fe in fish collected from the local markets January–April, May–August, and September–December 2022

Species	Cu				Zn				Fe			
	January-April	May-August	September-December	Average	January-April	May-August	September-December	Average	January-April	May-August	September-December	Average
Tilapia	0.70± 0.20 bA	0.14± 0.02 aB	0.75± 0.16 aA	0.53± 0.13 a	2.20± 0.47 eA	2.09± 0.12 cA	2.20± 0.25 efA	2.16± 0.28 e	1.54± 0.37 fA	1.46± .20 fA	1.34± 0.24 bA	1.45± 0.27 f
Catfish	0.33± 0.03 cA	0.11± 0.04 aB	0.30± 0.10 bA	0.25± 0.06b c	8.41± 0.68 aB	18.62± 1.60 aA	10.27± 1.50 aB	12.44± 1.26 a	9.33± 0.66 aB	12.10± 0.75 aA	8.41± 1.18 aC	9.50± 0.73 a
Common Carp	0.39± 0.10 cA	ND aB	ND cB	0.13± 0.01c de	3.34± 0.39 dA	ND eB	3.22± 0.70 deA	2.19± 0.62 e	7.72± 0.32 cA	2.37± 0.13 eB	1.10± 0.10 cC	3.73± 0.18 c
Roving Groupers	ND dA	ND aA	ND cA	ND d	3.50± 0.36 dcB	5.30± 0.47 bA	2.75± 0.35 defC	3.85± 0.32 c	3.50± 0.27 eA	3.00± 0.49 dB	ND dC	2.17± 0.53 e
Groupers	1.17± 0.10 aA	0.04± 0.05 aB	ND cB	0.40± 0.03 b	4.04± 0.46 cB	5.90± 0.30 bA	5.40± 0.66 bA	5.11± 0.47 b	9.09± 0.89 abA	4.86± 0.20 cB	1.65± 0.30b bcC	5.20± 0.46 b
Mackerels	ND eB	0.02± 0.02 aB	0.49± 0.04 bA	0.17± 0.01 cde	0.99± 0.02 dC	6.29± 0.26 bA	1.87± 0.25 fB	3.05± 0.18 d	5.20± 0.60 dA	2.65± 0.17 eB	ND dC	2.62± 0.26 d
Emperors	ND dA	ND aA	ND cA	ND d	4.50± 1.00 cA	4.90± 0.26 bA	4.70± 0.31 bcdA	4.70± 0.52 b	8.70± 0.90 bA	6.58± 0.11 bB	ND dC	5.09± 0.34 b
Silver Pomfret	0.36± 0.01 dA	ND aA	ND cA	0.12 ±0.0 e	1.99± 0.38 eC	4.37± 0.10 bA	3.78± 0.05 dB	3.38± 0.18 d	5.40± 0.81 dA	5.00± 0.88 cA	ND dC	3.47± 0.56 c

Data are reported as mean ± SD (n =4). Values with the same small letters in the same column are not significantly different, p< 0.05. Values with the same capital letters in the same raw are not significantly different, *p*< 0.05. Cu= Copper, Zn= Zinc, Fe= Iron.ND means not detected.

**Table 4:** Species and seasonal variations in levels (mg/kg fresh weight) of Co, Ni, and Mn in fish collected from the local markets January-April, May-August, and September-December 2022

Species	Cu				Ni				Mn			
	January-April	May-August	September-December	Average	January-April	May-August	September-December	Average	January-April	May-August	September-December	Average
Tilapia	0.70± 0.20 bA	0.14± 0.02 aB	0.75± 0.16 aA	0.53± 0.13 a	2.20± 0.47 eA	2.09± 0.12 cA	2.20± 0.25 efA	2.16± 0.28 e	1.54± 0.37 fA	1.46± .20 fA	1.34± 0.24 bA	1.45± 0.27 f
Catfish	0.33± 0.03 cA	0.11± 0.04 aB	0.30± 0.10 bA	0.25± 0.06b c	8.41± 0.68 aB	18.62± 1.60 aA	10.27± 1.50 aB	12.44± 1.26 a	9.33± 0.66 aB	12.10± 0.75 aA	8.41± 1.18 aC	9.50± 0.73 a
Common Carp	0.39± 0.10 cA	ND aB	ND cB	0.13± 0.01c de	3.34± 0.39 dA	ND eB	3.22± 0.70 deA	2.19± 0.62 e	7.72± 0.32 cA	2.37± 0.13 eB	1.10± 0.10 cC	3.73± 0.18 c
Roving Groupers	ND dA	ND aA	ND cA	ND d	3.50± 0.36 dcB	5.30± 0.47 bA	2.75± 0.35 defC	3.85± 0.32 c	3.50± 0.27 eA	3.00± 0.49 dB	ND dC	2.17± 0.53 e
Groupers	1.17± 0.10 aA	0.04± 0.05 aB	ND cB	0.40± 0.03 b	4.04± 0.46 cB	5.90± 0.30 bA	5.40± 0.66 bA	5.11± 0.47 b	9.09± 0.89 abA	4.86± 0.20 cB	1.65± 0.30b bcC	5.20± 0.46 b
Mackerels	ND eB	0.02± 0.02 aB	0.49± 0.04 bA	0.17± 0.01 cde	0.99± 0.02 dC	6.29± 0.26 bA	1.87± 0.25 fB	3.05± 0.18 d	5.20± 0.60 dA	2.65± 0.17 eB	ND dC	2.62± 0.26 d
Emperors	ND dA	ND aA	ND cA	ND d	4.50± 1.00 cA	4.90± 0.26 bA	4.70± 0.31 bcdA	4.70± 0.52 b	8.70± 0.90 bA	6.58± 0.11 bB	ND dC	5.09± 0.34 b
Silver Pomfret	0.36± 0.01 dA	ND aA	ND cA	0.12 ±0.0 e	1.99± 0.38 eC	4.37± 0.10 bA	3.78± 0.05 dB	3.38± 0.18 d	5.40± 0.81 dA	5.00± 0.88 cA	ND dC	3.47± 0.56 c

Data are reported as mean ± SD (n =4). Values with the same small letters in the same column are not significantly different, p< 0.05. Values with the same capital letters in the same raw are not significantly different, *p*< 0.05. Co= Cobalt, Ni= Nickel, Mn= Manganese.ND means not detected.

**Table 5:** Species and seasonal variations in levels (mg/kg fresh weight) of Cr, Cd, and Pb in fish collected from the local markets January-April, May-August, and September-December 2022

Species	Cr				Cd				Pb			
	January-April	May-August	September-December	Average	January-April	May-August	September-December	Average	January-April	May-August	September-December	Average
Tilapia	2.90± 0.17 deA	ND aB	0.02± 0.01 aB	0.97± 0.06 cd	ND aA	ND aA	ND aA	ND aA	3.49± 0.60 bA	ND aB	ND aB	1.16± 0.20 b
Catfish	2.21± 0.24 fA	ND aC	0.14± 0.09 aA	0.78± 0.12 de	ND aA	ND aA	ND aA	ND aA	2.03± 0.13 dA	ND aB	ND aB	0.68± 0.04 d
Common Carp	6.80± 0.10 aA	ND aB	ND aB	2.27± 0.03 a	ND aA	ND aA	ND aA	ND aA	1.70± 0.20 eA	ND aB	ND aB	0.57± 0.07 d
Roving Groupers	1.70± 0.17 gA	ND aB	ND aB	0.57± 0.05 e	ND aA	ND aA	ND aA	ND aA	5.05± 0.86 aA	ND aB	ND aB	1.68± 0.27 a
Groupers	3.18± 0.29 dA	ND aB	ND aB	1.06± 0.10 c	ND aA	ND aA	ND aA	ND aA	2.80± 0.35 cA	ND aB	ND aB	0.93± 0.22 c
Mackerels	4.33± 0.28 bA	ND aB	ND aB	1.44± 0.09 b	ND aA	ND aA	ND aA	ND aA	0.89± 0.14 fA	ND aB	ND aB	0.30± 0.05 e
Emperors	3.68± 0.20 cA	ND aC	0.14± 0.03 aB	1.27± 0.08 b	ND aA	ND aA	ND aA	ND aA	1.51± 0.06 eA	ND aB	ND aB	0.50± 0.02 d
Silver Pomfret	2.50± 0.37 efA	ND aB	0.09± 0.01 aB	0.86± 0.12 cd	ND aA	ND aA	ND aA	ND aA	1.69± 0.04 eA	ND aB	ND aB	0.56± 0.01 d

Table 3^3^ presents the seasonal variation in Cu, Zn, and Fe levels among the tested fish species. Tilapia showed the highest Cu concentration (0.53 mg/kg), whereas catfish exhibited the high-level contents of Zn (12.44 mg/kg) and Fe (9.50 mg/kg). Additionally, seasonal variations between the levels of Cu contents in most of the fish species collected either during January–April or September–December 2022 compared with samples collected during May–August. In the most cases, the levels of Cu in samples collected in January–April were higher than those obtained during May–August or September–December 2022.

Table 6 presents the maximum permissible limits (MPLs) recommended by international organizations, serving as a reference for assessing metal contamination in fish. The levels of Cu in all the tested fish species collected during the experiment as well as the average concentration levels did not exceed the acceptable limits (Table 6) [46–49].

**Table 6:** Maximum permissible limits (MPLs) recommended by national and international organization

	MPLs (mg/kg)								
Organization	Cu	Zn	Fe	Co	Ni	Mn	Cr	Cd	Pb
FAO (1983)	30	40					1		2.5
WHO (1989)	30	100		50			2	1	2
JECFA (2003)	3	100			0.5-1	1	0.15		2
USEPA (2000)	120	120					8	2	4
EC (2001)	3	100			0.5-1		1	1	1.5
EOSQ (2008 and 2010)								0.05	
EC (2014)		30				1	1	0.5	0.3
EC (2023)								0.05	

Cu is an essential element, playing a crucial role in the enzyme processes and the structure of hemoglobin [50]. In the current study, the levels of Cu were lower than those reported from different countries, namely, Bangladesh [51] and Saudi Arabia [52]. Although Cu is thought to be necessary for metabolic activity, large quantities are known to affect gastrointestinal, cardiovascular, hematological, hepatic, renal, and central nervous system functioning and may cause acute toxicity [53].

Regarding the levels of Zn, it was found that Catfish, Roving Grouper, Groupers, Mackerels, Emperors, and Silver Pomfret collected during May–August had higher levels of Zn compared to January–April and September–December 2022. The levels ranged from 2.16 to 12.44 mg/kg fresh weight, where the lowest and highest values were recorded for Tilapia and Catfish, respectively (Table 3). In this study, the average Zn concentrations in all seasons remained below the established acceptable limits (Table 6) [46–49].

Zinc (Zn) is an essential trace element mostly present in all organisms and acts an important role in metabolic processes. Nevertheless, excessive Zn levels in food can make people’s bodies deficient in Fe and Cu, resulting in symptoms including nausea, vomiting, fever, headaches, stomach pain, and skin irritation [54]. Also, data in Table 3 illustrate that all the tested fish species except Catfish collected during January–April had higher levels of Fe compared with samples collected during May–August and September–December 2022. The average concentration levels ranged from 1.45 to 9.50 mg/kg fresh weight, where the lower and higher values were recorded for Tilapia and Catfish, respectively. The levels of Fe in all the tested samples did not exceed the safe limit (Table 6) [46–49]. Iron is known to carry oxygen from the lungs to all the tissues, participate in enzyme systems in various tissues, play a significant role in fish growth and yield, and enhance protein and lipid metabolism in fish [55–57]. The high levels of Zn and Fe in fish, especially Tilapia and Catfish may be attributed to the addition of these metals to the diet and water in an aquaponic system through direct addition to the water, incorporation into fish feed, and foliar spraying of iron onto plant leaves [56, 58].

In January–April, higher levels of Ni were recorded in Catfish collected (6.34 mg/kg fresh weight), while Groupers had the lowest levels (1.07 mg/kg fresh weight) and were obtained in Tilapia (Table 4). In September–December 2022, the lower and higher levels were recorded for Common Carp (0.40 mg/kg fresh weight) and Emperors (3.80 mg/kg fresh weight), respectively. However, Ni was not detected in all the fish samples collected during May–August 2022. Although trace amount of Ni is necessary to activate some enzyme functions, and it is uncommon for Ni to induce toxicity for humans due to its absorption by the body being very low, high amounts of Ni can be hazardous to humans [59]. The levels of Ni in fish collected during January–April and September–December 2022, as well as the average concentration levels, were higher than the permissible limits requiring 0.5–1.0 mg/kg [48, 49]. The detected levels of Ni found in the investigated species were higher than reported by many investigators [51, 60], while these levels were found to be lower than those reported in water, sediments, and shrimp of Red Sea Coast of Jizan, Saudi Arabia [52]. Higher levels of Ni exposure via the food may cause many harmful health effects, including lung inflammation, fibrosis, emphysema, and clusters of aberrant skin cells [61].

Mn was not detected in all fish samples collected during January–April and September–December 2022, except Tilapia and Catfish collected during September–December 2022 (Table 4). However, Mn content was detected in May–August 2022 in all the investigated fish species, and Roving Grouper had the higher levels (10.0 mg/kg), while Common Carp had the lower levels of Mn (1.24 mg/kg). Mn is an essential micronutrient having a direct influence on numerous biochemical processes in the human body, such as metabolic pathways, including Mn-containing enzyme systems [61]. In the same way, the levels of Mn in fish collected in May–August 2022 as well as the average concentration levels in Roving Grouper and Mackerels were higher than the permissible limits requiring < 1.0 mg/kg [49, 62]. Also, the average concentration levels of Mn in the Mackerels and Roving Grouper were higher than the established maximum permissible limit. The current levels of Mn were found to be close to many studies [51, 60]. Several anthropogenic activities were reported to be sources of Mn, such as welding fuel addition, ferromanganese production, pesticides, and wastewater [54].

Data in Table 5 illustrate that Cd was not detected in all the tested seasons. Also, Cr and Pb were not detected in all samples collected during May–August 2022. However, seasonal variations in the levels of Cr and Pb in different species of fish were observed. In samples collected during May–August 2022, the higher content was recorded in Common Carp (6.80 mg/kg), while the lowest content was recorded for Roving Grouper (1.70 mg/kg). The levels of Cr in fish collected during May–August 2022 were higher than the permissible limits [46, 48, 49, 63]. The levels of Cr in all samples were lower than those from Jizan, Saudi Arabia [52], but they were comparable to those found in fish collected from the Bangshi River in Bangladesh [51] and Okumeshi River in Nigeria [60]. As is well recognized, Cr is a necessary component for numerous human and animal processes and aids in the body’s lipid metabolism [61, 64]. On the other hand, Cr may cause cancer and damage to the kidney, liver, and brain [65–67]. Cd is the fifth most dangerous heavy metal, and the burning of coal, fertilizers, insecticides, detergents, and refined petroleum products is the main way that cadmium enters the environment [68]. The body accumulates cadmium, a very hazardous heavy metal that is not necessary [11]. According to the standards, fish should have no more than 0.05 mg/kg of Cd [69–71]. In this study, all the analyzed samples had non-detectable amounts of Cd that were below the quantification limit (Table 5). The upper limit of Cd concentration in mussels intended for human consumption was set to be 1 mg/kg fresh weight [48]. The current findings are consistent with those of Okyere et al., who revealed that fish collected from Ghana’s Muni Lagoon had cadmium levels below the detection limit [72]. Also, samples collected either during May–August or September–December 2022 contained non-detectable levels of Pb, while all the analyzed samples collected during January–April had detectable amounts of lead quantification, and Mackerels had the lower Pb content (0.89 mg/kg), while Roving Groupers had the high-level contents (5.05 mg/kg). All the analyzed samples collected during January–April exceeded the maximum permissible limit of Pb established in the guidelines for fish [62]. Pb is a highly hazardous and non-essential metal that pollutes the environment and harms human health by damaging the kidney, brain, neurological system, red blood cells, and bones [73]. Industrial operations, gasoline, paints, water pipes, batteries, contaminated drinking water and foods, and tobacco use are the main causes of lead exposure [74]. Furthermore, the current findings demonstrated that there were no consistent patterns of seasonal fluctuations in fish metal concentrations over the course of the testing periods. The current findings are consistent with Rajeshkumar et al. who found that there was little evidence of a seasonal effect on the concentration of metals in fish tissue [75].

In the present study, the concentrations of the most tested metals (Cu, Fe, Co, Cr, and Pb) during the wet season (January–April) were higher than the other tested periods, while the concentrations of Zn and Mn were higher during the period May–August compared to the other tested periods. The higher levels of Cu, Fe, Co, Cr, and Pb during the wet season might be attributed to the increased rainfall during the wet season can lead to greater metal runoff from industrial, agricultural, and urban sources; this influx raises metal concentrations in water, leading to higher bioaccumulation in fish [76–78]. The absence of Ni in May–August samples may be due to the bioaccumulation of Ni by fish and their sensitivity to Ni were modified by temperature, suggesting that seasonal variations are likely to occur. During summer, stronger water currents and thermal stratification may dilute metal concentrations, reducing their bioavailability to fish [79]. The distribution of eight heavy metals (Fe, Mn, Cu, Zn, Cr, Ni, Pb, and Cd) in three species namely *Pinctada radiata*, *Brachidontes pharaonic*, and *Holothuria polii* was determined seasonally from seven stations along Alexandria Coast, Mediterranean Sea, Egypt [80]. The highest annual averages for each of the eight investigated metals were found in *Pinctada radiata*, while the lowest averages were found in *Holothuria polii* for Fe, Zn, Pb, Cr, Cu, Ni, and Cd, and in *Brachidontes pharaonis* for Mn (3.405 μg/g wet weight). The findings of the present study agree with that published about the relation between the concentration of metals and the seasonal variation [81]. The highest levels of Zn, Pb, Cu, Co, Fe, Mn, and Ni recorded in autumn and winter might suggest that there was an inverse relation with phytoplankton productivity [82] and the slower growth rate at cooler temperatures [83]. When the availability of food is lower, the reserve is used for metabolism leading to a decrease in the body mass and an increase in the metal concentration [84]. The season with the high-level contents of metals can vary depending on the specific location and various factors, such as the levels of metals in water and food, water chemistry, and the species, size, age, gender, feeding habits, habitat, pH, temperature, and physiological status of fish [58, 78, 85–88]. Compared to the pre-summer season, the post-monsoon season in India seems to have the high-level contents [26]. Additionally, the warm layer traps the air close to the earth’s surface during the winter, preventing pollutants from escaping the atmosphere and ultimately leading to their accumulation [26]. However, during the summer, air from the atmosphere rises and combines with the clean air, enhancing the dispersion of pollutants over a large area [89]. There was little evidence of a seasonal effect in concentration. Fish gathered from the Yenisei River in Russia showed minor fluctuations within and between years for Zn, K, Na, Ca, and Mg, but considerable irregular variations in the levels of Fe, Mn, Cu, Ni, Pb, Co, and Cr were investigated [90]. However, the metal accumulation trend peaked in the summer and then continued throughout the fall, winter, and spring was recorded [19]. Variable levels of agricultural runoff and the release of untreated household and industrial waste into rivers and oceans could be the cause of these variations [91]. Overall, the current findings are consistent with those of numerous researchers who found that the average concentrations of Cd, Cr, Cu, Zn, and Pb in the muscle of *S. pursakensis* and *C. carpio* obtained from the Kızılırmak river and Damsa Dam Lake, Turkey, are below the allowable limits [58, 86].

### Correlation Matrix Between the Tested Elements

The Pearson correlation coefficient was used to assess whether there was a correlation between metal levels. The values of Pearson’s correlation of several metals in fish muscles at the 0.05 and 0.01 levels of significance are presented in Table 7. The correlation coefficients are classified based on strength [92] as follows: very weak (*r* = 0.0–0.2), slightly significant (*r* = 0.2–0.4), moderate (*r* = 0.4–0.6), strong (*r* = 0.6–0.8), and profound (0.8–1.0).

**Table 7:** Pearson correlation among heavy metals present in s muscles of different fish species

	Cu	Zn	Fe	Co	Ni	Mn	Cr	Cd	Pb
Cu	1								
Zn	0.079	1							
Fe	0.075	0.079	1						
Co	− 0.134	0.932**	− 0.441	1					
Ni	− 0.717*	− 0.171	0.306	0.406	1				
Mn	− 0.390	0.460	− 0.414	0.294	0.559	1			
Cr	0.407	.379	0.407	0.268	0.375	0.386			
Cd	1**	1**	1**	1**	1**	1**	1**		
Pb	0.280	− 0.190	0.389	0.387	0.406	0.401	− 0.507*	1	1

^*^, **Correlations are significant at the 0.05 and 0.01 levels, respectively (2-tailed)

In Table 7, it was shown that there was a profound positive relationship for Cd and all the tested metals (1.00) and for Zn/C (0.932), which is significant at the 1% level of significance. Moderate positive relationships were observed for Mn/Ni (0.559), Mn/Zn (0.460), Cr/Fe (0.407), Ni/Co (0.406), Pb/Ni (0.406), and Pb/Mn (0.401). Additionally, slightly significant relationships were recorded for Pb/Fe (0.389), Pb/Co (0.387), Cr/Mn (0.386), Cr/Cu (0.379), Cr/Ni (0.375), Ni/Fe (0.306), Mn/Co (0.294), and Co (0.268), while very weak relationships were obtained for Zn/Cu (0.079), Zn/Fe (0.079), and Fe/Cu (0.075). This suggests that the concentrations of these metals in fish may be interrelated, possibly due to similar accumulation pathways within fish organisms [34]. This implies that the concentrations of these metals in fish might be interrelated, perhaps because of comparable accumulation mechanisms within fish organisms [34]. These findings may explain the possibility of metal–metal interactions that could occur under the environmental concentrations. However, a statistically significant negative correlation was observed for Ni/Cu (− 0.717), Cr/Pb (0.507), Co/Fe (0.4441), Mn/Fe (0.414), and Mn/Cu (− 0.390) at *p* < 0.01. Also, feebly negative relations were observed for Co/Cu (− 0.134), Ni/Zn (− 0.171), and Pb/Zn (− 0.190). The present results are in parallel with many investigators who reported that positive correlations among Ni, Cu, Zn, Cd, and Pb [92, 93], while a non-linear relationship was observed with other elements [94]. It was suggested that strong correlations among metals might reflect a common origin and similar sources, which can be related to geological structure in the studied region, while negative correlations might indicate differing geochemical behaviors and external influences [95–97]. Exposure to more than one pollutant may cause antagonistic, synergistic, additive, and/or interactive effects [98].

### Metal Pollution Index (MPI)

The level of total metal accumulation and the extent to which heavy metal pollution is present in different fish species was compared using the MPI (Table 8), where MPI can be used in monitoring programs to provide information on the bioavailability, bioconcentration, and environmental input of metals [99]. A higher MPI value indicates that the sample has accumulated more metal aggregates, making it an accurate indicator of metal pollution in food samples [100]. Consumption of fish with a high MPI value may pose a potential public health risk [85]. The MPI values were < 2, 2–5, 5–10, 50–100, and > 100, indicating no, very low, low, high, and extremely contamination, respectively [101, 102]. Freshwater fish exhibited MPI values between 1.36 and 1.52, with the highest in Catfish, indicating potential contamination. In contrast, marine fish showed lower MPI values (1.40–1.58), suggesting minimal health risks. MPI of metals were found to range from 0.381 to 2.216 in *Pomatomus saltatrix* and *Scomberomorus commerson* species collected from Alexandria beaches, South-Eastern Mediterranean Sea, Egypt [103]. These findings highlight the importance of monitoring metal accumulation to ensure food safety. The variation in the concentrations of metals and sizes may be species-specific, depending on habitats, food and feeding patterns, metabolic processes, and water qualities [8]. Also, the differences in metal bioconcentration were attributed to diverse metal bioavailability in water and the dissimilar ways fish can cope with each metal, including inefficient excretion mechanisms [104]. A higher value of MPI reflects the greater gross accumulation of metals in fish [102]. The bioaccumulation and/or bioconcentration of metals in fish is a complex process that depends on the physiology of the species and the bioavailability of metals in water, which depends on water physicochemical properties such as alkalinity, temperature, and pH [58, 104]. The eating habits, detoxifying ability, gill absorption activity and rate, and consumption of contaminated food are all associated with fish physiology [105]. Metals are known to bioaccumulate/bioconcentrate in many organs, including gills, liver, intestine, and kidney, as well as muscles [104]. Muscles were found to have low bioconcentration values of most metals compared with those for the gills or liver [106] indicating that bioaccumulation is related to tissue structure [107]. The metal will be transported by the bloodstream to muscles when the metal concentration exceeds the visceral detoxification capacity, resulting in its bioaccumulation in the muscles [108]. The presence of metals in muscles is an indication of chronic exposure [109]. The bioaccumulation of these metals in different fish was associated with metals in water and sediment [110]. The presence of metals in fish can lead to risks for consumers [111, 112]. Although metals are bioconcentrated more in the viscera than the muscles [104], in the present study, the consumption of these contaminated muscles with these metals may pose a threat to Alexandrian peoples who live close to lakes, rivers, and seas. As a result, muscles may be a good way to measure long-term exposure to heavy metals, and exposure to such metals, along with seasonal variations, might influence their bioaccumulation in fish tissues [10].

**Table 8:** Metal pollution index and health risk parameters for metals found in the fish studied

Metal	Metal Pollution Index	EDI (µ/kg body weight/day)	ADI (µ/kg body weight/day)	Hazard Index (EDI/ADI, %)	Reference dose * (µ/kg body weight/day)	THQ
Cu	1.36	0.20	2800	7.14 × 10^−5^	40	5.00 × 10^−3^
Zn	1.52	4.68	21,000	2.22 × 10^−4^	300	1.56 × 10^−2^
Fe	1.38	4.21	49,000	8.60 × 10^−5^	700	6.01 × 10^−3^
Co	1.58	2.48	280	8.68 × 10^−3^	20	1.24 × 10^−1^
Ni	1.41	1.75	1400	1.25 × 10^−3^	20	8.75 × 10^−2^
Mn	1.40	1.20	9800	1.22 × 10^−4^	140	8.57 × 10^−3^
Cr	1.49	1.17	210	5.57 × 10^−3^	1500	7.80 × 10^−4^
Cd	1.46	0.00	7	0.00	1.0	0.00
Pb	1.36	0.81	105	7.71 × 10^−3^	1.50	5.40 × 10^−1^

^*^According to USEPA [43]

### Human Health Risk Assessment

The human health risk posed by metals via the consumption of fish was assessed based on the estimated daily intake (EDI, µg/kg/day), hazard index (HI, %), and the target hazard quotient (THQ, %) of the selected elements.

### Estimated Daily Intake (EDI)

The estimated daily intake (EDI) values (µg/kg bw/day) for the tested metals (Cu, Zn, Fe, Co, Ni, Mn, Cr, Cd, and Pb) with their corresponding acceptable daily intake, which probably will not bring any noticeable lifelong risks (Table 8). The risk associated with EDI of various fish species, based on the target hazard quotient (THQ), is shown in Table 8. THQ is a measure used to assess non-carcinogenic health risks from metal exposure. The mean EDI values of metals followed an ascending order of Cd < Cu < Pb < Cr < Mn < Ni < Co < Fe < Zn and ranged between 0.0 and 4.68. All tested metals exhibited EDI values lower than the reference doses [43], indicating that consuming these fish poses no significant health risk for adults [104], where the hazard index values (EDI/ADI,%) for the metals analyzed were Cd: 0.0, Cu: 7.14 × 10⁻^3^, Fe: 8.60 × 10⁻^5^, Mn: 1.22 × 10⁻^4^, Zn: 2.22 × 10⁻^4^, Ni: 1.25 × 10⁻^3^, Cr: 5.57 × 10⁻^3^, Pb: 7.71 × 10⁻^3^, and Co: 7.71 × 10⁻^3^. Given that the metals assessed and the fish species under investigation are safe to eat, it is unlikely that fish intake will have an impact on sensitive populations.

### Target Hazard Quotient (THQ)

The hazard quotient (HQ) and hazard index (HI) were used for determining the non-carcinogenic risk when consuming fish, where the non-carcinogenic risk refers to the possibility of occurrence of negative effects on the human health. HQ is a proportion of the probable exposure to an element or chemical at such a level with no expected negative impacts, while HI is a vital index that assesses overall likely impacts associated with exposure to more than one contaminant [113]. The consequences of non-carcinogenic risk are related to the occurrence of harmful health impacts represented by negative reactions, dysfunction of the human body to the manifestation of different diseases of the neuro, cardiovascular systems, and all organs, except cancer [114, 115]. The THQ ratio aids in gauging the potential health impacts from metal intake from eating fish. The exposed population is unlikely to be at risk if the THQ ratio is < 1, while when THQ is ≥ 1, there is a potential health risk associated with exposure to metals via fish consumption [116, 117].

In the current study, the calculated THQ values of metals followed an ascending order of Cd < Cr < Cu < Fe < Mn < Zn < Ni < Co < Pb and ranged between 0.0 and 0.54 (Table 8). The data illustrate that the THQ of all the tested metals in muscles of the investigated fish species are below 1 suggesting that consumption of these fish species is unlikely to cause non-carcinogenic health effects resulting from the intake of these metals. The THQ values for Mn, Cu, Zn, Cr, Pb, and Cd in fish collected along Alexandria Coast, Mediterranean Sea, Egypt, were found to be close to that obtained in the present study, indicating that there is no human risk from their consumption [80]. Also, the non-carcinogenic health risk from the present study aligned with many studies [93, 118]. On the other hand, the THQ values of Cd in fish collected from Manzalah Lake, Egypt were found to be higher than one [103].

Food safety is especially crucial for vulnerable populations, such as children, the elderly, and those with weakened immune systems, particularly expectant mothers and nursing mothers [119, 120], children are more susceptible to heavy metals than adults, and the presence of high levels of metals has been linked to increase the risk of developing a variety of diseases, including those affecting the nervous, cardiovascular, and digestive systems [121, 122]. High levels of spontaneous abortions, stillbirths, premature deliveries, decreased birth weights and infant lengths, newborn behavioral problems, and other adverse outcomes have been linked to pregnant women’s exposure to heavy metals [123]. Pb exposure causes mental diseases in children, including learning difficulties, mental health issues, and mental agitation. It also raises the levels of hemolytic anemia [124, 125]. Also, Pb damage causes minimal ripening, the death of conception, unrestricted fetal removal, neurological, cardiovascular, and gastrointestinal disorder, as well as mutagenic and cancer-causing effects [125]. Lower exposure to Co might cause skin rashes, nausea, and vomiting, while prolonged exposure might cause diarrhea, bleeding, coma, and even death [125]. In postmenopausal women, Cd had a similarly detrimental effect on bone density [126]. In addition to visual and hearing loss, low-level Cd exposure can cause harm to the kidneys, liver, bones, and cardiovascular system. In addition to the potent teratogenic and mutagenic effects associated with cadmium, cadmium adversely affects pregnancy or its outcome [126, 127]. Additionally, Mn may cause impairments in children’s neurobehavior and cognitive function [128, 129].

## Conclusion

The results indicated seasonal and species variation in the levels of the tested metals. Also, the levels of Cu, Zn, Fe, Co, and Cd in all the tested fish species collected during the experiment did not exceed the guideline limits, while Ni, Cr, and Pb in in fish collected during January–April and September–December, May–August, and January–April 2022, respectively, had higher levels than the permissible limits settled by the national international organizations. Additionally, Mackerels and Roving Groupers had higher levels of Mn. The accumulation of these metals in muscles of different fish species has a wide range reflecting the variation in the accumulation of these elements. As found in the present study, the average concentration levels regarding the tested metals showed that the most predominant elements were Cu and Co in Tilapia, Zn in Catfish, Roving Groupers, and Mackerels, Fe in Common Carp, Groupers, Emperors, and Silver Pomfret. The most abundance metals in fish were Fe, Zn, and Co. MPI of the metals in the investigated species ranged from 1.36 to 1.58. The obtained EDI, HI, and THQ values were found to be less than the units, indicating that there is no potential health risk for Alexandrian people associated with exposure to the tested metals via the consumption of these fish species. It is recommended that human exposure to metals from food should be restricted to the lowest levels by implementing strict specifications limiting the quantities in the food. The present study recommends to consider the concentration limits of toxic metals set by the national and international organizations, because these metals may accumulate in various parts of fish over an extended period and cause health risk to consumers. Also, these findings insights into the urgent need for monitoring, management, and focusing on the heavy metal sequestration from industrial waste streams and to create a scientific framework for restricting heavy metal discharges into the aquatic environment.

## Limitations of This Study

Data provided in this research article is on the muscles of the fishes investigated, which is the part principally consumed by Alexandrians. Future studies will consider additional tissues (e.g., liver, gills) to give a more complete understanding of the bioaccumulation of metals in fish. Also, the health risk assessment was also conducted only for adults; therefore, future research on different age groups and more sensitive populations (children, women, and pregnant women) will be considered. The present study included a limited variety of fish species; therefore, a wide range of fish species will be taken into consideration for investigation. These limitations must be acknowledged because they present an opportunity for future research to fill research gaps and improve the understanding of the quality of fish for consumers in the studied area.

## Data Availability

No datasets were generated or analysed during the current study.
