# Correction: Marketing of Freshwater and Marine Fish Species in Alexandria City, Egypt: Human Health Risk of Specific Metals

**DOI:** 10.1007/s12011-025-04667-1

**Published:** 2025-07-12

**Authors:** Khaled A. Osman, Hala H. Elsayed Mohamed, Maher S. Salama

**Affiliations:** 1 Pesticide Chemistry and Technology Department, Faculty of Agriculture, EL-Shatby, Alexandria University, Aflaton St., EL-Shatby, Alexandria, 21545 Egypt; 2 The Faculty of Organic Agriculture, Heliopolis University, P.O. Box 2834, Cairo, Egypt

**Correction: Biological Trace Element Research**

10.1007/s12011-025-04596-z

The published version of this article unfortunately contained a mistake.

In this article Hala H. Elsayed Mohamed and Maher S. Salama at affiliation ‘The Faculty of Organic Agriculture, Heliopolis University, P.O. Box 2834, Cairo, Egypt and Pesticide Chemistry and Technology Department, Faculty of Agriculture, EL-Shatby, Alexandria University, Aflaton St., EL-Shatby, 21545 Alexandria, Egypt’ were missing from the author list.

The original article has been corrected.

